# Radiomics and artificial intelligence applications in pediatric brain tumors

**DOI:** 10.1007/s12519-024-00823-0

**Published:** 2024-06-27

**Authors:** Francesco Pacchiano, Mario Tortora, Chiara Doneda, Giana Izzo, Filippo Arrigoni, Lorenzo Ugga, Renato Cuocolo, Cecilia Parazzini, Andrea Righini, Arturo Brunetti

**Affiliations:** 1 Department of Precision Medicine, University of Campania “L. Vanvitelli”, Caserta, Italy; 2 Department of Advanced Biomedical Sciences, University of Naples “Federico II”, Via Pansini 5, 80131 Naples, Italy; 3 Department of Head and Neck, Neuroradiology Unit, AORN Moscati, Avellino, Italy; 4 Department of Pediatric Radiology and Neuroradiology, V. Buzzi Children’s Hospital, Milan, Italy; 5 Department of Medicine, Surgery and Dentistry, University of Salerno, Baronissi, Italy

**Keywords:** Machine learning, Neuroradiology, Pediatric brain tumors, Radiomics

## Abstract

**Background:**

The study of central nervous system (CNS) tumors is particularly relevant in the pediatric population because of their relatively high frequency in this demographic and the significant impact on disease- and treatment-related morbidity and mortality. While both morphological and non-morphological magnetic resonance imaging techniques can give important information concerning tumor characterization, grading, and patient prognosis, increasing evidence in recent years has highlighted the need for personalized treatment and the development of quantitative imaging parameters that can predict the nature of the lesion and its possible evolution. For this purpose, radiomics and the use of artificial intelligence software, aimed at obtaining valuable data from images beyond mere visual observation, are gaining increasing importance. This brief review illustrates the current state of the art of this new imaging approach and its contributions to understanding CNS tumors in children.

**Data sources:**

We searched the PubMed, Scopus, and Web of Science databases using the following key search terms: (“radiomics” AND/OR “artificial intelligence”) AND (“pediatric AND brain tumors”). Basic and clinical research literature related to the above key research terms, i.e., studies assessing the key factors, challenges, or problems of using radiomics and artificial intelligence in pediatric brain tumors management, was collected.

**Results:**

A total of 63 articles were included. The included ones were published between 2008 and 2024. Central nervous tumors are crucial in pediatrics due to their high frequency and impact on disease and treatment. MRI serves as the cornerstone of neuroimaging, providing cellular, vascular, and functional information in addition to morphological features for brain malignancies. Radiomics can provide a quantitative approach to medical imaging analysis, aimed at increasing the information obtainable from the pixels/voxel grey-level values and their interrelationships. The “radiomic workflow” involves a series of iterative steps for reproducible and consistent extraction of imaging data. These steps include image acquisition for tumor segmentation, feature extraction, and feature selection. Finally, the selected features, via training predictive model (CNN), are used to test the final model.

**Conclusions:**

In the field of personalized medicine, the application of radiomics and artificial intelligence (AI) algorithms brings up new and significant possibilities. Neuroimaging yields enormous amounts of data that are significantly more than what can be gained from visual studies that radiologists can undertake on their own. Thus, new partnerships with other specialized experts, such as big data analysts and AI specialists, are desperately needed. We believe that radiomics and AI algorithms have the potential to move beyond their restricted use in research to clinical applications in the diagnosis, treatment, and follow-up of pediatric patients with brain tumors, despite the limitations set out.

**Graphical abstract:**

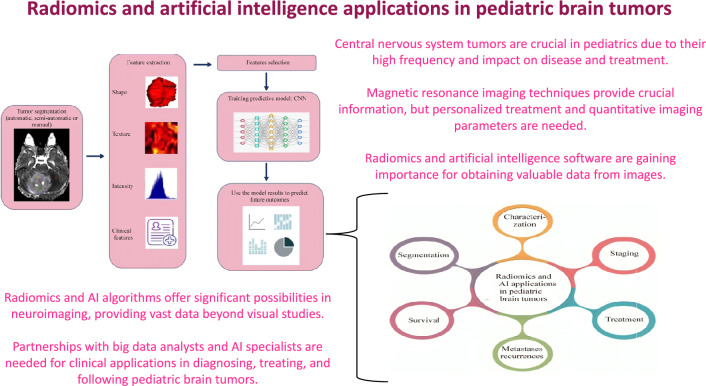

## Introduction

Central nervous system (CNS) tumors are the most common solid tumors in children. The incidence of CNS tumors in children and young adults is about 6 in 100,000 individuals [1]. The 2021 World Health Organization (WHO) classification of CNS tumors (WHO CNS5) incorporated molecular data alongside with histology, leading to several changes including the subdivision of pediatric- and adult-type gliomas based on different mutation pathways [2, 3]. Low-grade gliomas (LGGs) represent the most common pediatric CNS (p-CNS) tumor, constituting about 18% of all pediatric neoplasms. Pilocytic astrocytoma (PA) represents the most common subgroup of LGGs. Other less common LGGs include pilomyxoid astrocytoma, pleomorphic xanthoastrocytoma, diffuse astrocytoma, dysembryoplastic neuroepithelial tumor, and subependymal giant cell astrocytoma [1, 4]. Molecular alterations in LGGs involve the RAS–mitogen-activated protein kinase (RAS–MAPK) pathway, which ultimately leads to stimulation of cell proliferation. LGGs are WHO grade I/II tumors that can arise anywhere along the neuroaxis, but most commonly involve the infratentorial region [4]. High-grade gliomas (HGGs) in children range between 15 and 20% of p-CNS tumors. The most common HGGs include anaplastic astrocytoma (WHO grade III), glioblastoma (WHO grade IV), and diffuse intrinsic pontine glioma (DIPG) [1]. Another important p-CNS tumor group comprises embryonal tumors, among which medulloblastoma (MB) (WHO grade IV) represents the most common neoplasm, accounting for about 10% of all p-CNS tumors [4]. Genetic studies have resulted in a classification of medulloblastoma into 4 subgroups: WNT-activated, Sonic hedgehog-activated (SHH), and groups 3 and 4 which are less defined but mainly related to the amplification of the MYC gene and the instability of chromosome 17 [4].

Ependymoma (EP) represents about 5% of p-CNS. These tumors typically occur intracranially in the posterior fossa (PF) but can also arise in the supratentorial region or along the spine. EP can be classified as WHO grade I (including myxopapillary EP and subependymoma), grade II (classic EP), or grade III (anaplastic EP) [1].

Genetic analysis has led to a classification system of EP based on the identification of the RELA fusion protein, in which the mutation leads to constitutive activation of the nuclear factor kB (NF-Kb) pathway, and on the identification of *YAP1* (transcription regulator of genes involved in cell proliferation) fusion with other genes [4]. The WHO 2021 classification of p-CNS tumors has included genetic alterations in the grading system, indicating that understanding the neoplasmic genetic basis is important in advancing therapeutic developments, better understanding tumor behavior, predicting its response to different treatments, and developing new therapies, thereby moving towards an increasingly personalized approach. Magnetic resonance imaging (MRI) techniques are typically capable of differentiating between LGG and HGG neoplasms, combining morphological (lesion localization, signal intensity, tissue components, and post-contrast behavior) and non-morphological (diffusion and perfusion information) tumor characteristics. However, accurately determining histological typing and defining the neoplasm’s natural behavior might be difficult to assess using only MRI qualitative parameters due to the extreme tumor variability, which is also dependent on different genotypes (Table 1).

**Table 1:** Overview of pediatric glioma regarding types, key genetic features with percentage and general incidence

p-LGG type	Key genetic features	Genomic percentage	General incidence
Circumscribed astrocytic gliomas			
Pilocytic astrocytoma	*KIAA1549-BRAF, BRAF, NF1*	*KIAA1549-BRAF* (70%–80%) *FGFR1-TACC1* (3%–5%) *FGFR1* SNV (3%–5%) *BRAF* p.V600E (3%–5%) [45]	Cerebellar PA: 15%–25% of all pediatric CNS tumors [46]
Subependymal giant cell astrocytoma (SEGA)	*TSC1, TSC2*	*TSC1/2* SNV (85%–95%) [45]	~ 10% of tuberous sclerosis complex patients [47]
Pleomorphic xanthoastrocytoma	*BRAF, CDKN2A/B*	*BRAF* p.V600E (80%–90%) [45]	< 1% of all astrocytomas [48]
Chordoid glioma	*PRKCA*	*PRKCA* SNV (80%–90%) [45]	Rare, few cases reported [49]
High-grade astrocytoma with piloid features	*BRAF, NF1, ATRX, CDKN2A/B* (methylome)	Deletions of *CDKN2A/B* ~ 80%; alterations of MAPK pathway ~ 75%; ATRX ~ 45%; MGMT promoter methylation ~ 45% [50]	Rare, few cases reported (~ 80% of a histologically defined anaplastic pilocytic astrocytomas) [50]
Astroblastoma *MN1*-altered	*MN1*	*MN1* break-apart noted in 62.5% of a cohort of 8 patients; *MN1* monosomy in one case and intact in two cases [51]	0.45%–2.8% of all gliomas [51]
Pediatric-type diffuse low-grade gliomas			
Diffuse astrocytoma, *MYB*- or *MYBL1*-altered	*MYB, MYBL1*	*MYBL1* alteration (5%–10%) [45]	2% of p-LGGs [52]
Angiocentric glioma	*MYB*	*MYB* (80%–90%) [45]	Rare, few cases reported [53]
Polymorphous low-grade neuroepithelial tumor of the young (PLNTY)	*BRAF, FGFR* family	*BRAF* p.V600E (30%–40%) *FGFR2/3* fusions (30%–40%) [45]	Rare, few cases reported [54]
Diffuse low-grade glioma, MAPK pathway-altered	*FGFR1, BRAF*	Most common *BRAF* V600E mutations and *FGFR1* alterations either a duplication or mutation in the tyrosine kinase domain [55]	9% of all p-LGG [56]
Diffuse midline glioma, H3 K27-altered	H3 K27, *TP53, ACVR1, PDGFRA, EGFR, EZHIP*	*ACVR* 1–32%; *TP53* ~ 60–80%; *PDGFRA* ~ 30%; *EGFR* ~ 4%; *NF1* ~ 10%; *MYC* ~ 12%; *ATRX* ~ 10% [57]	15–20% of all brain tumors in pediatric population [58]
Diffuse hemispheric glioma, H3 G34-mutant	H3 G34, *TP53, ATRX*	Concurrent *TP53* and *ATRX* ∼90% of cases; *MGMT* promoter is frequently methylated. Between 50 and 70% harbour *PDGFRA* mutations [59]	About twice less frequent than H3.3 K27M mutations [60]
Diffuse pediatric-type high-grade glioma, H3-wildtype and *IDH*-wildtype	IDH-wildtype, H3-wildtype, *PDGFRA*, *MYCN*, *EGFR* (methylome)	~ 50% *MYCN* amplification in pedGBM_MYCN subgroup; ~ 22% *CDK4/6* amplification in pedGBM_MYCN subgroup; ~ 33% *PDGFRA* amplification in pedGBM_RTK1 subgroup; ~ 50% *EGFR* in pedGBM_RTK2 subgroup [61]	Estimated to represent a large proportion (about 40%) of p-HGG with p-HGG MYCN and p-HGG RTK2 [59]
Infant-type hemispheric gliomas	*NTRK* family, *ALK, ROS, MET*	Fusion of *NRTK* gene present in 10% of non-brainstem p-HGG [62]	Rare, few cases reported [62, 63]

*p-LGG* pediatric low-grade gliomas, *H3* histone 3, *K* lysine, *G* glycine, *SNV* single nuclear variation, *PA* pilocytic astrocytoma, *CNS* central nervous system

Radiomics is an area of research applying a quantitative approach to medical imaging analysis, aimed at increasing the information obtainable from the pixels/voxel grey-level values and their interrelationships. The simplest descriptors are 1-order texture features, that are based on histogram analysis, while the second-order descriptors also include information on the spatial interrelationship between pixel/voxel grey-level values (Table 2) [5]. The huge amount of data that can be mined from medical images with radiomics can then be analyzed by machine learning (ML) algorithms.

**Table 2:** A summarized view of histogram and texture features

Histogram features	Texture features
Mean (median gray intensity within the ROI)	Gray-level co-occurrence (GLCM) (matrix which quantifies the spatial relationships of pixel intensities in an image)
Maximum (maximum gray intensity within the ROI)	Gray-level run-length (GLRLM) (matrix which describe the distribution of consecutive pixels with the same intensity value in a specific direction)
Minimum (minimum gray intensity within the ROI)	Gray-level size zone (GLSZM) (matrix which describes the size and intensity distribution of connected regions which share the same gray intensity
Variance (mean of the squared distances of the values from the mean)	Gray-level distance zone (GLDZM) (matrix which describes the size and intensity distribution of connected regions which share the same gray intensity requiring them to be at the same distance from the ROI edge)
Percentiles (percentiles of the values)	Neighborhood gray-tone difference (NGTDM) (matrix which quantifies the sum of differences between the intensities in a pixel or voxel by the mean of a neighbor pixel or voxel within a defined distance)
Skewness (asymmetry of the data distribution curve)	Neighborhood gray-level dependence (NGLDM) (matrix which quantifies the sum of differences between the intensities in a pixel or voxel by the mean of a neighbor pixel or voxel within a defined distance requiring them to be in a defined range of gray intensities differences)
Kurtosis (deviation of a data distribution from a Gaussian distribution attributed to the presence of outliers)	
Entropy (randomness in an image value)	
Energy (measure the magnitude of voxel values in an image)	

**Fig. 1:**
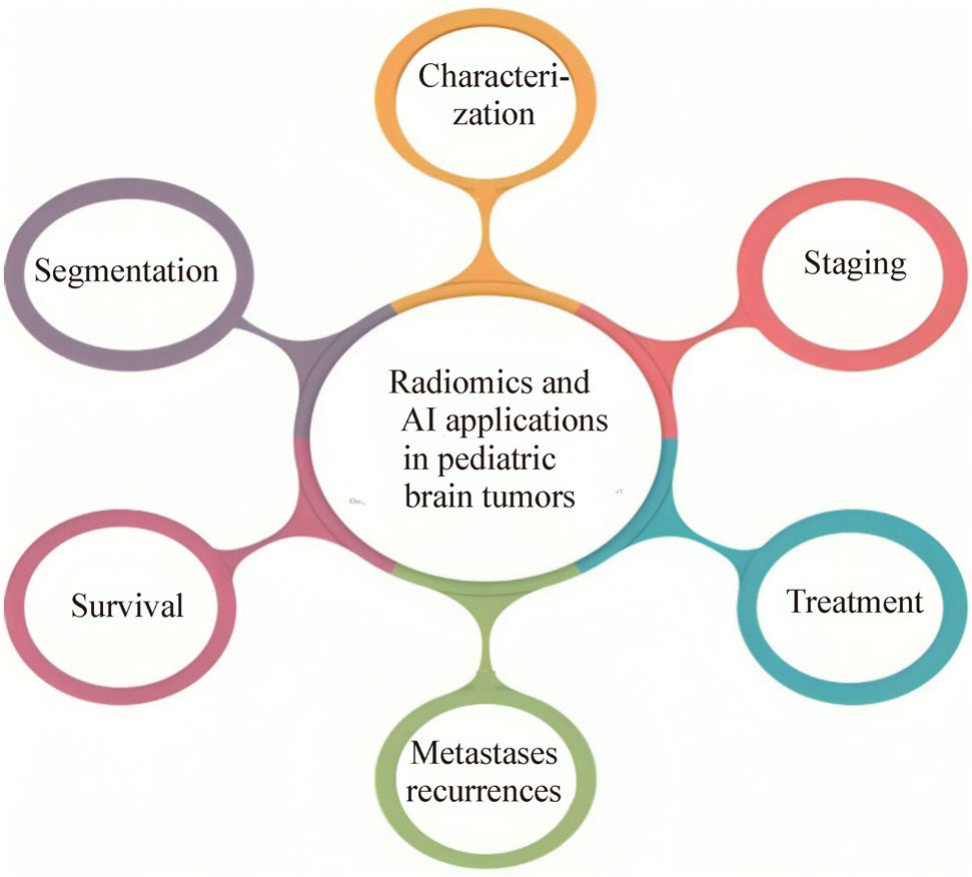
The “radiomic workflow” involves a series of iterative steps for reproducible and consistent extraction of imaging data. These steps include image acquisition for tumor segmentation, feature extraction, and feature selection. Finally, the selected features, via training predictive model (CNN), are used to test the final model

Figure 1 depicts a radiomic workflow, which involves a series of iterative steps for achieving reproducible and consistent extraction of imaging data. These steps include image acquisition for tumor segmentation, feature extraction, and feature selection. Finally, the selected features, via training predictive models such as vector machines, random forests, logistic regression, and k-nearest neighbors, are used to test the final model [6].

**Fig. 2:**
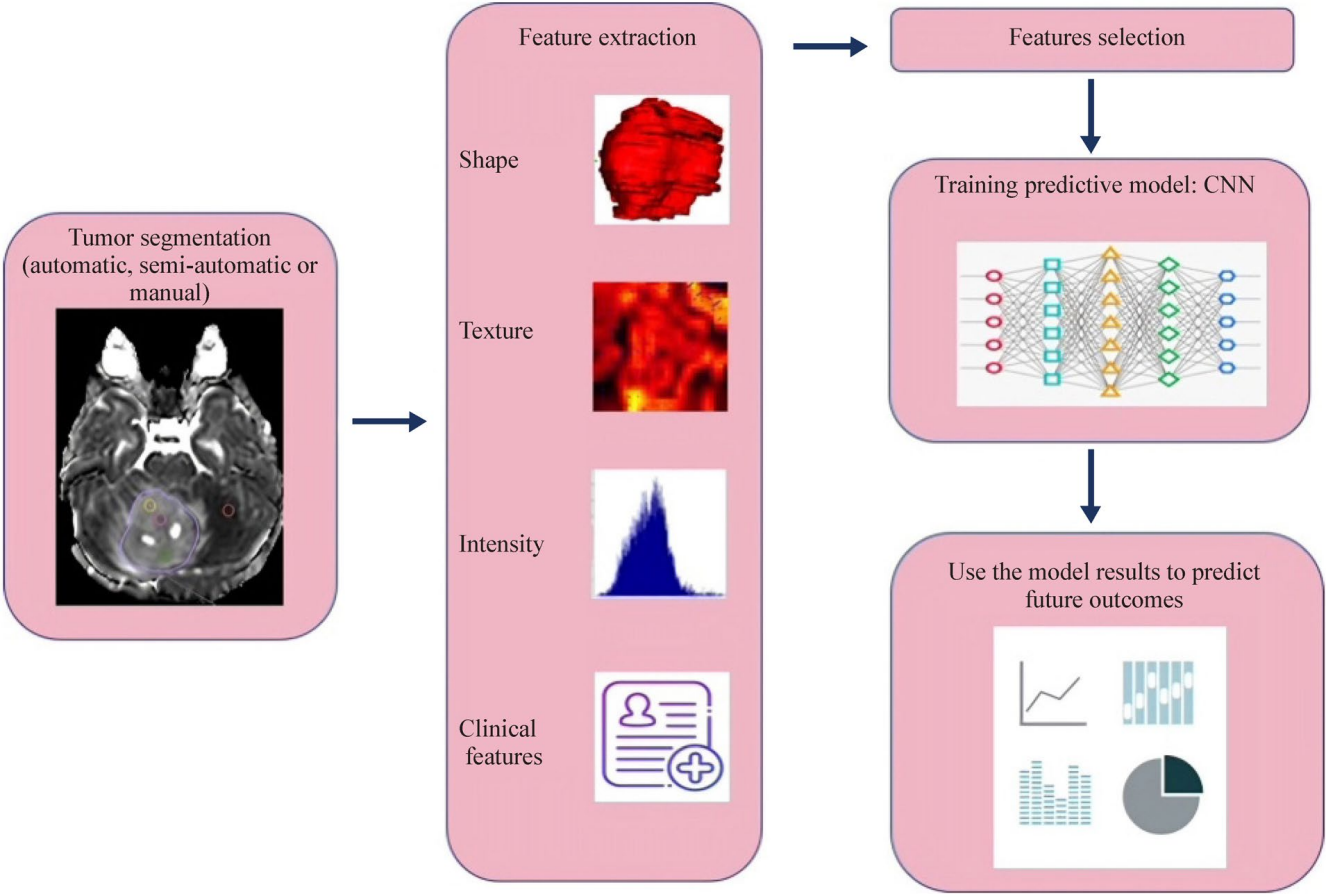
The main steps of a radiomic workflow (segmentation and characterization) to obtain predictions of staging, survival, metastasis and recurrence, and treatment responses

In this review, we present an overview of radiomics and machine learning studies, focusing on the different research areas in which these techniques can be implemented on p-CNS tumors, such as lesion segmentation, grading, differential diagnosis, prediction of prognosis, evaluation of treatment response, and prediction. The present limitations of radiomics will also be discussed. The outline of our paper is shown in Fig. 2.

## Conventional magnetic resonance imaging sequences for diagnosis

The most prevalent solid tumor in children and a major cause of death for this demographic are primary brain tumors. Imaging is essential for brain tumor diagnosis, characterization, treatment planning, and disease surveillance. MRI serves as the cornerstone of neuroimaging, providing cellular, vascular, and functional information in addition to morphological features for brain malignancies. When paired with demographics and clinical presentation, imaging findings can aid in the development of a precise diagnosis or a limited differential diagnosis [7]. Pre- and post-contrast T1-weighted, T2-weighted, fluid-attenuated inversion recovery (FLAIR), and diffusion-weighted sequences are all part of a standard brain MRI. In T1-weighted imaging, the use of high-resolution, thin-section, three-dimensional images that are flexible in several planes has become standard practice and is becoming more widely accepted for T2- and FLAIR-weighted sequences as well. In a previous article, it was found to be particularly capable of differentiating between equivalent healthy tissue, tumor tissue, and edema in T2 sequences. This capability provides support for the identification and delineation of tumor burdens and informs the development of decision trees or radiomic algorithms for diagnostic purposes and multimodal management, including surgery and radiotherapy [8].

Furthermore, technological developments in imaging methods, such as parallel imaging and compressed sensing, enable the capture of thinner, high-resolution slices with acceptable scan times. Our clinical practice research group follows a standard operating procedure that is based on working groups for response assessment in pediatric neuro-oncology (RAPNO). These groups have released recommendations for the minimally necessary MRI sequences for various kinds of pediatric brain cancers [9–12] (Table 3). The mobility of water molecules is measured by diffusion-weighted images and is influenced by the complexity of cytoarchitecture. Reduced apparent diffusion coefficient (ADC) values indicate limited diffusion in high-grade cancers, which may be quantitatively assessed due to their increased cellularity and high nuclear-to-cytoplasmic ratios [13]. In the case of non-enhancing tumors in particular, diffusion-weighted imaging is beneficial for both leptomeningeal metastasis identification and differential diagnosis of brain cancers [14]. Standard brain imaging sometimes includes susceptibility-weighted imaging (SWI), which is particularly sensitive to paramagnetic or diamagnetic substances and can identify regions of bleeding or calcifications [15].

**Table 3:** Overview of pediatric brain tumors with common locations and key MRI features

Tumor type	Tumor sub-type	Common location	Key MRI features
Embryonal tumors	Medulloblastoma	Exclusively posterior fossa: fourth ventricle or cerebellar vermis (non-WNT, non-SHH, or WNT); cerebello-pontine angle (WNT) or cerebellar hemispheres (SHH)	Diffusion restricting Variable enhancement Cystic/necrotic change Calcifications uncommon Taurine peak characteristic
	Atypical teratoid/rhabdoid tumor	Posterior fossa or cerebral hemispheres	Diffusion restricting Enhancement usually present and more heterogenous than medulloblastomas, with cysts/necrosis, calcification, and hemorrhage
	Supratentorial embryonal	Cerebral hemispheres or deep nuclei	Large tumor Diffusion restricting solid components Variable cysts/necrosis change and hemorrhage
Glial and glioneuronal tumors	Diffuse midline glioma (H3K27 altered)	Ventral pons and thalami	Expansile, ill-defined mass No diffusion restriction Usually, non-enhancing at presentation
	Supratentorial high-grade glioma (H3 G34 mutant or H3 wildtype)	Hemispheric (frontal and parietal lobes) or deep nuclei	Large, circumscribed tumor Diffusion restricting solid components Variable cysts/necrosis and hemorrhage
	Pilocytic astrocytoma	Cerebellum (common), brainstem, optic chiasm/hypothalamus	Cyst with enhancing mural nodule; may be completely solid
	Posterior fossa ependymoma	Fourth ventricle or cerebello-pontine angle	Heterogeneous mass Calcifications common Intermediate diffusion (between medulloblastoma and pilocytic astrocytoma) Usually enhancing High myo-inositol
	Supratentorial ependymoma	Frontal or parietal lobes	Large mass with necrosis Central chunky calcifications Diffusion restriction
	Ganglioglioma	Temporal lobe	cystic with enhancing mural nodule No diffusion restriction
	Dysembryoplastic neuroepithelial tumor (DNET)	Frontal or temporal lobes, cortically based	Well-circumscribed, triangular configuration, ‘bubbly’ appearance Absent or minimal enhancement May be associated with cortical dysplasia Scalloping of the overlying calvarium
Pineal region and choroid plexus tumors	Pineoblastoma	Pineal region	Heterogenous, ‘explode’ pineal calcification Diffusion restricting Enhancement variable
	Germ cell tumor	Pineal and/or suprasellar region	Heterogenous, usually calcified, ‘engulf’ pineal calcification Diffusion restricting Enhancement present
	Choroid plexus papilloma	Lateral ventricle followed by fourth ventricle	Lobular or papillary lesions Diffuse enhancement No diffusion restriction High myo-inositol, low creatine, moderate choline
	Choroid plexus carcinoma	Lateral ventricle	More heterogenous than papillomas Parenchymal invasion/edema Diffusion restriction Moderate myo-inositol and high choline

*MRI* magnetic resonance imaging, *SHH* Sonic hedgehog-activated, *H3* histone 3, *K* lysine, *G* glycine

## Advanced magnetic resonance imaging sequences for diagnosis

Information on microstructure, hemodynamics, and metabolism may be obtained by advanced imaging methods. Diffusion tensor imaging (DTI) may be post-processed to produce white matter tracts and evaluate the direction and amount of water diffusion. They aid in directing surgical techniques and offer insightful information on the tumor’s proximity to important white matter tracts [16]. Tumor vasculature and hemodynamics can be evaluated using MRI perfusion methods. Contrast methods, such as dynamic susceptibility (DSC), dynamic contrast-enhanced perfusion (DCE), and contrast-free arterial spin labeling (ASL), can be used to accomplish perfusion. Hemodynamic measures, such as relative cerebral blood flow (rCBF), relative cerebral blood volume (rCBV), time to peak (TTP), mean transit time (MTT), and vascular permeability or transfer coefficient (K-trans), can be obtained from perfusion MRI, depending on the technique employed. These data can offer insightful information on the tumor’s grade, response to therapy, and ability to distinguish radiation damage from tumors [16]. Tumor categorization and differential diagnosis can benefit from the examination of tissue biochemical composition provided by magnetic resonance spectroscopy (MRS) [17].

## Segmentation

Lesion segmentation plays a key role in the radiomic workflow and is a step that can be performed using a region of interest (ROI) or a volume of interest (VOI). Furthermore, segmentation can be manual, automatic, or semi-automatic. Manual segmentation is usually an exceedingly long process and prone to inter-individual variability, while semi-automatic segmentation is initiated by the operator and is then operated by an algorithm according to various strategies such as clustering, region-growing, active contours, and watershed transformation [18]. Alternatively, a semi-automated ROI/VOI mask can also be produced by manual editing to correct the output of an automated segmentation tool. Semi-automatic segmentation, compared to the entirely manual approach, is also susceptible to variability based on reader bias. Kalpathy-Cramer et al. evaluated the reproducibility of lung nodule segmentations by comparing measures obtained from three different segmentation algorithms in a dataset of 52 tumors. The agreement in the measures was significantly higher in repeated runs of the same algorithm than when compared to different algorithms. Moreover, the variability of results was also associated with the initial ROI provided by the operator [18, 19]. Fully automatic segmentation has the advantage of being fast; however, its main limitation is its susceptibility to false positives and false negatives [18]. Some authors have used ML to calculate tumor volumes in children. For example, Peng et al. used processed images from pre- and post-operative MRIs, which included T1 contrast-enhanced (T1-CE) and T2/FLAIR images of p-HGGs, MBs, and leptomeningeal seeding tumors. These were used to train a three-dimensional (3D) U-Net Neural Network architecture to automatically segment the lesions and an algorithm to automatically score the product of the maximum bidimensional diameters according to the response assessment in pediatric neuro-oncology (RAPNO) criteria. Their automated pipeline showed high agreement with human segmentation for both pre-operative and post-operative models, as well as for the attribution of RAPNO scores [20].

At present, the most effective method may still be the use of semi-automatic segmentation, which allows the expert operator to refine the results provided by the algorithm. However, the standardization of a uniform method and algorithm has yet to be achieved (Table 4).

**Table 4:** Overview of study characteristics (divided by topic)

Articles	Number of patients	Subsite	Imaging	Analyzed endpoint	Statistical findings	Conclusions
Zhou et al., Automatic machine learning to differentiate pediatric posterior fossa tumors on routine MR imaging	288	PF tumors (MB EP, PA)	MRI: T1w, T2w, ADC maps	Characterization	3087 radiomics features	Tree-based pipeline optimization tool models achieved higher accuracy than average qualitative expert MRI review (0.83 versus 0.54, *P* < 0.001)
Zhang et al., Radiomics can distinguish pediatric supratentorial embryonal tumors, high-grade gliomas, and ependymomas	231	Supratentorial tumours: HGGs, EPs, embryonal tumours	MRI: T1w, T2w	Characterization	1800 radiomic features	LR had the best accuracy in embryonal tumours vs HGGs (AUC = 0.98), XGB in embryonal tumours vs. EPs (AUC = 0.82), neural net in HGGs vs. EPs (AUC = 0.96)
Li et al., Ependymoma and pilocytic astrocytoma: differentiation using radiomics approach based on machine learning	45	PF tumors (EP vs PA)	MRI	Characterization	300 radiomic features including texture, wavelet and Gabor transform features	Texture features contribute the most in the differentiation
Jie et al., Differentiation between ependymoma and medulloblastoma in children with radiomics approach	51	PF tumors (EP vs MB)	MRI: T1w post-contrast, ADC maps	Characterization	188 radiomic features	Logistic regression as feature selection method and random forest as classifier achieved the highest result (AUC = 0.91)
Novak et al., Classification of paediatric brain tumours by diffusion-weighted imaging and machine learning	117	PF tumors (EP vs MB vs PA)	MRI: ADC maps	Characterization	Histogram features: min, max, mean, median, variance, skew, Kurtosis and the quartiles	Random forest model classification showed the highest accuracy = 86.3%; suggested ADC cutoff between MBs and EPs of 0.984 × 10 − 3 mm2 s − 1; a significant difference between the mean tumour values in EPs vs. PAs vs. MBs *P* < 0.001
Grist et al., Distinguishing between pediatric brain tumor types using multi-parametric magnetic resonance imaging and machine learning: a multi-site study	49	PF tumors (EP vs MB vs PA) EPs and MBs considered high-grade, PA low-grade	MRI:DWI and dynamic susceptibility contrast imaging (DSC)	Characterization	Image mean, standard deviation, skewness, and kurtosis on ROI and whole brain	ADC mean had highest AUC = 0.8 for discrimination, AdaBoost classifiers highest performance (precision = 85%),
Iv M. et al., MR Imaging–based radiomic signatures of distinct molecular subgroups of medulloblastoma	109	MBs	MRI: T2w and T1w post-contrast	Molecular classification	590 radiomic features including: intensity-based histograms, tumor edge sharpness, Gabor features, and local area integral invariant (LAII)	Good performance of the SVM model using tenfold cross-validation in predicting SHH, group 3 and group 4 tumors (AUC 0.79, 0.70, and 0.83, respectively), not strongly predictive of WNT tumors (AUC 0.45–0.63)
Haldar et al., Unsupervised machine learning using K-means identifies radiomic subgroups of pediatric low-grade gliomas that correlate with key molecular markers	157	Low-grade gliomas	MRI: T1w pre- and post- contrast, T2w, T2 FLAIR	Molecular classification	881 radiomic features	Unsupervised approach through k-means clustering, which divided the cohort in three clusters. Of ten genes of interest: only BRAF was found to be statistically different in the groups (*P* = 0.0346)
Zheng et al., Clinical-MRI radiomics enables the prediction of pre-operative cerebral spinal fluid dissemination in children with medulloblastoma	84	MBs	MRI: T1w post-contrast	CSF dissemination prediction	385 radiomic features and clinical data	The multivariable logistic regression model which combined radiomic features and clinical data had the highest performance for predicting pre-operative CSF dissemination (AUC = 0.89 in the training cohort) vs 0.67 using clinical features alone
Jared H. Hara BS; Clinical applications of quantitative 3-dimensional MRI analysis for pediatric embryonal brain tumors	34	Embryonal brain tumors	MRI: T1w post-contrast and T2 FLAIR	Association to recurrence and metastatic disease	15 radiomic features	FLAIR and T1 post-contrast features such as “maximal tumor diameter”, “neighborhood gray tone coarseness”, and “strength” were associated with metastatic disease; FLAIR features such as “neighborhood gray-level co-occurrence matrix contrast”, “dissimilarity”, and “neighborhood gray tone contrast” were associated with metastatic disease; results not significant on univariate analysis
Pisapia et al.; Predicting pediatric optic pathway glioma progression using advanced magnetic resonance image analysis and machine learning	38	Optic pathway gliomas (OPG)	MRI: T1w post-contrast, T2w, T2-FLAIR, diffusion tensor imaging (DTI)	Prediction of progression	Intensity distributions were obtained from within the segmented regions on all imaging sequences, including derivatives of DTI	Features linked to Fractional anisotropy (FA) and T2w such as “higher intensity of FA within the OR”, “mean FA values within the OR”, “mean Radial diffusivity (RAD) within the OR”, and “mid distribution T2 intensity within ORs” were associated to progression
Grist et al.; Combining multi-site magnetic resonance imaging with machine learning predicts survival in pediatric brain tumors	69	p-CNS	MRI: ADC maps, DSC	Prognosis	Image mean, standard deviation, skewness and kurtosis calculated for ADC and uCBV/CCBV/K2 maps on ROI and whole brain; clinical data	Unsupervised k-means clustering divided the cohort in high- and low-risk groups, the hazard ratio was higher in the high-risk group, a univariate analysis showed some features were statistically different in the two groups; a supervised ML model had the highest accuracy in classification between high- and low-risk groups

*PF* posterior fossa, *MB* medulloblastoma, *EP* ependymoma, *PA* pilocytic astrocytoma, *HGGs* high-grade gliomas, *p-CNS* pediatric central nervous system, *MRI* magnetic resonance imaging, *ADC* apparent diffusion coefficient, DWI Diffusion Weight Imaging, *FLAIR* fluid-attenuated inversion recovery, *ROI* region of interest, uCBV uncorrect Cerebral Blood Volume and cCBV correct Cerebral Blood Volume, *LR* logistic regression, *AUC* area under curve, *XGB* extreme gradient boosting, *SVM* support vector machine, *OR* optic radiation, *ML* machine learning

## Characterization

The extraction of radiomic characteristics allows a large amount of data to be collected. When combined with other information, such as clinical and/or demographic characteristics, these data enable the development of tumor characterization models through ML algorithms. These models sometimes perform better than qualitative analyses conducted by expert readers [21]. This is particularly important for tumors of PF in which imaging characteristics alone may not allow a robust differential diagnosis. Zhou et al. compared the performance of ML models based on the 3-Based Pipeline Optimization Tool (TPOT) with models based on manual expert optimization and with qualitative expert MRI readings to characterize PF tumors (MB vs. EP vs. PA) by retrospectively evaluating 288 patients with these neoplasms. A total of 3087 radiomic features were extracted from pre-operative MRI, which included T1-CE, T2-weighted sequences, and ADC maps. Different models based on TPOT and manual expert optimization were then built in both multiclass and binary classifications of the tumors and compared with two expert qualitative readings. Overall, for multiclass classification, the best TPOT model achieved higher accuracy when compared with the average expert MRI review, which was also higher than the best model based on manual expert optimization, although not statistically significant. Both the best models based on TPOT and manual expert optimization tool performed similarly in binary classification; the TPOT model performed significantly better when compared to the expert’s qualitative reading [21].

To differentiate pediatric supratentorial embryonal tumors, HGGs, and EPs, Zhang et al. retrospectively evaluated 231 patients with supratentorial embryonal tumors, HGGs, and EPs, and from each tumor volume, they extracted 1800 radiomic features by T2- and T1-weighted imaging, which then underwent sparse regression analysis by a least absolute shrinkage and selection operator (LASSO). The reduced set of radiomic features with clinical variables, such as age at diagnosis and sex, was then used as input to train six different classifiers: support vector machine (SVM), logistic regression (LR), k-nearest neighbor, random forest (RF), extreme gradient boosting (XGB), and neural net. Both multiple binary classifiers and multiclass classifiers were then evaluated. For embryonal tumors vs. HGGs, 23 features were identified: the top three were age (clinical feature), T2-cluster shade (Gray Level Co-occurrence Matrix: GLCM), and T2-mean (first-order intensity); LR had the highest performance in this binary group. For embryonal tumors vs. EPs, XGB had the best performance where the top three relevant features included T2-kurtosis (first order), T1-informational measure of correlation (GLCM), and T1-skewness. For EPs vs. HGGs, neural net showed the highest performance with the top three relevant features including T1-mean (1-order intensity), T1-cluster shade (GLCM), and T2-maximal correlation coefficient. The performance of single tumor classifiers was lower than that of the binary classifiers [22].

To differentiate between EPs and PAs, Li et al. enrolled 45 patients with PF tumors. They extrapolated about 300 multimodal features, which were divided into three groups: (1) texture-based features, (2) Gabor transform-based features, and (3) wavelet transform-based features. Texture-based features collect quantizable parameters of the interrelationship between pixels, while Gabor and wavelet-based features are transformation-based features (higher order features encoding structural and frequency-based information of an image). Using a KWT filter-based method (a supervised method that evaluates each feature to remove invalid ones), they highlighted 80 multimodal features as significant (67.50% texture features). They trained an SVM classification system to differentiate between PAs and EPs. Among this set of features, the major contribution was from the texture features. Regarding the result of PAs vs. EPs, the overall feature set reached an excellent sensitivity [23].

Dong et al. evaluated which radiomic features could help to differentiate between EP and MB. In their cohort of 51 patients, they segmented the tumor volume on T1-CE images and then overlapped the VOI to ADC maps; they extracted 188 radiomic features from T1-CE images and ADC maps, which were normalized, reduced, and selected through different techniques like univariable analysis (UA) and multivariable logistic regression (MLR). The subset of features was then analyzed through four classifiers: k-nearest neighbor, adaptive boosting (AdaBoost), RF, and SVM; tenfold cross-validation was reiterated ten times on the cohort of study, which was divided into ten subgroups (the models took turns dividing nine parts as training data and one part as testing data in each reiteration). The optimal performance was yielded by a radiomics model built with the MLR feature selection method and with an RF classifier, which gained the highest AUC and accuracy. This model achieved an excellent sensitivity and precision for EPs and MBs. Moreover, four features had significant differences in EP vs. MB, which were spherical disproportion, median (ADC), information measure of correlation (T1C), and low gray-level zone emphasis (ADC) [24].

Novak et al. evaluated the use of extracted histogram parameters by ADC maps (including mean, variance, skew, and kurtosis) to help differentiate between PA, MB, and EP. They employed two different classification models, namely Naïve Bayes (NB) and RF. This combined model showed the highest performance with an overall classification accuracy. Observing that there was a significant mean tumor value difference in their study, they suggested a cut-off value between the MBs and EPs of 0.984 × 10^−3^ mm^2^ s^−1^ with high sensitivity and specificity [25]. Grist et al. combined multi-center diffusion and perfusion imaging (DSC-MRI) to develop an ML-based classifier aimed at distinguishing between EP, MB, and PA in a cohort of 49 patients. Significant differences were observed between PA and MB in terms of ADC ROI mean, ADC ROI skewness, and ADC ROI kurtosis, and in whole-brain features such as corrected cerebral blood volume (CBV) mean and ADC mean. Additionally, significant differences were found between tumor volumes of PA and EP. In their study between the ML classifiers, the best result was achieved using a combination of all the ROI features with an SVM [26] (Table 4). Currently, research on radiomic and pediatric neuro-oncological pathology emphasizes the pivotal role of developing models that leverage radiomic characteristics for tumor characterization, as indicated by the included articles. Despite the potential of these algorithms to perform at par or even surpass human readers, there is a recognized need to optimize this approach [27]. To achieve this, it is important to establish a standardized model for radiomic characteristics, focusing on the differential diagnosis that requires evaluation, thereby ensuring a thorough analysis. Moreover, the identification of the most effective ML model for analyzing these variables is essential for the successful integration of this technology into clinical practice.

## Staging and molecular classification

Molecular classification is a key element in the evaluation of the p-CNS. The WHO classification has incorporated these elements, emphasizing that the identification of some genetic patterns is pivotal for the implementation of the right therapeutic procedure. It would be valuable if some elements of radiomics could correlate with tumor mutations. Some studies have addressed this possibility. Iv et al. evaluated the radiomics features that could best correlate with the molecular subgroups of 109 patients with MBs. They compared two validation schemes for predicting the tumor subgroups: a double tenfold cross-validation on a single dataset, which contained all three patient cohorts included in the study, and a 3-dataset cross-validation, involving training on 2 cohorts and testing on a third independent cohort. From an initial set of 590 MRI radiomic features extracted from T2-weighted and T1-CE, a group of features was selected through a Wilcoxon rank sum test, and then, an SVM classifier was used with a tenfold cross-validation strategy to predict molecular subgroups (WNT, SHH, group 3, and group 4). The model with the tenfold cross-validation strategy on a single dataset performed well in predicting the SHH, group 3, and group 4 tumors, particularly when using extracted quantitative data from both T1- and T2-weighted images. On the other hand, the three-dataset cross-validation scheme resulted in good performance in discriminating SHH and group. The four leading feature categories were lesion area, edge sharpness, local area integral invariant (LAII), and histogram. However, both schemes performed comparatively less robustly in predicting WNT and group 3 subgroups [28].

To assess which radiomic features in patients with LGGs could better correlate with molecular signatures, Haldar et al. evaluated 157 subjects with a diagnosis of pediatric LGG (p-LGG) in an unsupervised approach. A total of 881 MR radiomic features were extracted by pre- and T1-CE sequences, T2-FLAIR, and T2-weighted sequences, which were then z-scored. A feature reduction was performed using Principal Component Analysis (PCA) to reduce the dimension of the dataset; the 48 elements that accounted for 90% of the variance were then considered for the clustering step. K-means clustering was performed with 10,000 iterations on the selected PCA components, identifying three clusters of patients with similar feature profiles. A Chi-squared test was then applied to determine significant differences between variables such as gene mutations (mutated vs. wild type). Only BRAF exhibited a significantly different mutational frequency across the three clusters. In addition, age, tumor location, and tumor histology differed significantly between the clusters [29].

Finally, it is important to note that WHO CNS5 2021 includes specific glioma mutations that can be identified with quantitative biomarkers. These have proven to be useful for grade determination and molecular definition (IDH status) of gliomas. These biomarkers can be extrapolated from both conventional MR parameters Visually AcceSAble Rembrandt Images (VASARI score) [30] and advanced MR parameters such as spectroscopy [31] (Table 4).

## Neuro-oncologic radiomics: from research to clinical workflow

Imaging parameters, particularly ADC and perfusion mapping, alongside FLAIR, T2-weighted, and T1-CE images, combined with radiomics and deep learning techniques (e.g., Convolutional neural networks: CNN), have the potential to greatly assist radiological assessments in neuro-oncology. Imaging is crucial to the management of brain tumors, for treatment and prognosis [32]. Indeed, predicting outcomes and tracking therapy responses are 2 of the primary objectives of radiomics research in cancer. Leading neuro-oncology publications have debated and published prospective uses for radiomics. For instance, a recent study on diffuse midline gliomas (DMGs) demonstrated how radiomic features extracted from T1 and T2 MRI sequences correlated with statistical significance with progression-free survival (PFS) and overall survival (OS). Diagnostic performance tests showed a specificity for PFS and a sensitivity greater than 90%. For OS, sensitivity ranged from 80 to 90% [33]. However, these uses, particularly in pediatric oncology, have yet to be implemented in clinical practice. The rarity of many brain tumors could be the cause of this. Radiomics must fill gaps in the literature or enhance current circumstances before it can be integrated into clinical practice.

## Treatment

Treatment of p-CNS can range from single modality to multi-modality, including surgery, radiotherapy, and systemic therapy, depending not only on the tumor itself but also on other factors such as age or clinical presentation. Surgery still has a particularly key role, not only for tumors that can be completely excised but also as a method of debulking—reducing tumor size in patients eligible for treatment using different modalities [34]. For example, in the case of p-HGGs, management is maximally safe surgical resection followed by focal radiation therapy. However, the discovery of mutation pathways has led to clinical trials investigating the use of target therapy, such as histone deacetylase inhibitors and tyrosine kinase inhibitors [1]. The management of MBs includes safe surgical resection followed by risk-adapted craniospinal irradiation (CSI) and adjuvant chemotherapy in children over 3 years old. Risk assessment in patients with MBs takes into account factors such as age, metastatic disease, and size of residual tumor. Subsequently, high- and low-risk categories are identified, which influences the radiation dose to be administered. Low-risk patients receive 23.4 Gy, while high-risk patients receive 36 Gy, with an additional boost of 54 Gy to the tumor bed in both cases [1].

Some tumor spreading may be missed at MRI, such as the dissemination of the MB in the CSF, and it may be challenging to repeatedly perform CSF tests. To assess which radiomic feature could be more predictive of dissemination and therefore influence the prognosis of these patients, Zheng et al. studied 124 cases of MBs including 44 cases with CSF dissemination, comparing a mixed model of multivariable analysis of clinical/radiomic features vs. one clinical model. From the MRIs of these patients, 385 radiomic features were extracted from pre-operative T1-CE images, which included histogram parameters, volume, and shape parameters, as well as Haralick features, gray-level co-occurrence parameters, and gray-level run-length matrix parameters. The top nine radiomic features that were best associated with dissemination were then selected using minimum redundancy and maximum correlation (mRMR) and LASSO during the training phase. The model was then validated on two validation cohorts (internal and external). The mixed model, which included nine radiomic features and one clinical, had an AUC better than for the clinical model alone. A decision curve analysis (DCA) showed that at every probability threshold, it was more beneficial to apply the mixed model instead of the clinical model. Given these findings, some radiomic features may be used to reduce the risk of including in the low-risk category patients with underestimated or undiagnosed CSF dissemination at qualitative assessment alone. This can assist in preventing the administration of under-dosed radiotherapy with a lower response to treatment [35] (Table 4).

## Metastases, recurrence, and progression

Hara et al. retrospectively evaluated the MRI examinations of 34 patients with embryonal brain tumors to study which radiomic features could better correlate with metastatic disease and recurrence. During the follow-up period, nine patients experienced recurrence, and six died. After tumors were delineated with ROI by a single operator from pre-operative T1-CE and FLAIR images, the data were exported to MATLAB 2017a where the number of radiomic features generated was kept close to the number of patients included. Under the review of a physicist and a neuroradiologist, 35 radiomic features were generated with known prognostic value for pediatric embryonal tumors. Among the initial 35 features, those with the largest observed variance were retained, which led to a subset of 15 radiomic features for statistical analysis. Cox regression was performed to evaluate the ability of these features to correlate with recurrence and survival outcomes, while logistic regression was used to assess their association with metastasis.

The authors demonstrated that FLAIR and T1-CE features, such as tumor size, decrease in primary tumor heterogeneity, and strength, were associated with metastatic disease. Other features extracted from FLAIR, such as neighborhood gray-level co-occurrence matrix contrast and dissimilarity, as well as neighborhood gray-tone contrast, resulted in being related to metastatic disease status. These results, however, were not statistically significant in logistic regression [36, 37]. Features in FLAIR related to size maximum 3D diameter and volume, and to decreased heterogeneity, such as neighborhood gray-tone coarseness, contrast, and busyness were associated with recurrence. Additionally, zone max entropy from T1-CE correlated with recurrence. Despite these associations, these correlations were not significant in the univariable analysis, most likely due to the small sample size [19].

A study by Pisapia et al., to obtain a predictive model of the progression of optic pathway gliomas (OPG), included 19 patients with OPG progression (characterized by a reduction of visual acuity or tumor size increase) and 19 control cases without progression. They used an ML model for the analysis of radiomic features extracted through a first manual segmentation of the optic nerves from the T1-CE images and a segmentation on diffusion tensor imaging (DTI) of the optical radiations. These segmented regions were then overlaid on all anatomic sequences and DTI sequences, including T1, T1-CE, T2, T2-FLAIR, fractional anisotropy (FA), radial diffusivity (RAD), and trace (TR) through a process of registration. The distribution of intensities within these regions, the minimum, maximum, mean, and standard deviation of values, for all MRI modalities, were then obtained. The extracted data were processed using an SVM through 2 analyses. In the 1 analysis, the ML model was used to cluster the patients based on the best subdivision of features that correlated with progression. These were then added to the model until no further accuracy was achieved. In the second analysis, the dynamic changes of features through a combination of follow-up MRI examinations were included as additional features in the model. Features that were more predictive of progression were linked to FA and T2. These included higher intensity of FA within the optic radiation (OR), mean FA values within the OR, mean RAD within the OR, and mid-distribution T2 intensity within the OR. Their highest ML model reached an accuracy about 90%. Out of the top ten features related to the progression of OPG, nine were related to DTI, an element that is consistent with white matter biological changes in gliomas. Moreover, their study showed the importance of adding dynamic features changing in predictive models [37, 38] (Table 4).

## Survival and prognosis

p-CNS tumors have a variable outcome dependent on the histological origins of the tumor and numerous other factors, with a wide range of survival rates based on histotype and grading. For instance, WNT MB has a > 95% 5 year survival rate [39, 40], group 3 MB has a 5 year survival rate of about 50% [39, 41], and brain stem gliomas have a median survival rate of 9 months [41]. To study which radiomic features could be predictive of the outcome of patients with p-CNS, Grist et al. analyzed radiomic features related to ADC maps and perfusion (DSC-MRI) in a cohort of 69 patients. For each imaging feature, they performed a univariable statistical analysis which showed that there were significant differences in whole-brain and ROI imaging features between high-grade and low-grade tumors. By performing Cox regression for each imaging feature, clinical data, and tumor grade, they calculated the survival hazard coefficients and, in a subsequent iterative Bayesian survival analysis, identified the top five imaging features that could better relate to survival, which included uncorrected Cerebral Blood Volume (uCBV) ROI mean, K2 ROI mean, uCBV whole-brain mean, tumor volume, and ADC ROI kurtosis. With unsupervised k-means clustering, two groups were created (high and low risk) in which a Kaplan–Meier analysis showed a significant statistical difference. A Cox regression showed a higher hazard ratio (HR) in the high-risk group than in the low-risk group. A univariable analysis performed on the features of the two groups highlighted a statistically significant difference in features such as ROI ADC kurtosis, ROI ADC skewness, ROI K2 mean, ROI CBV uncorrected standard deviation, K2 whole-brain standard deviation, and CBV corrected whole-brain mean. A supervised ML model using the features from the Bayesian analysis and a single layer of neurons reached the best classification accuracy (98% vs. 90% of logistic regression). It is interesting to note that the Kaplan–Meier analysis found differences in survival between the high-grade tumors included in the high-risk group and the low-risk group [42] (Table 4).

## Current limitations of radiomics studies

At present, the use of radiomic analysis techniques has some limitations that cannot be ignored. The wide variability of vendors and their distinctive characteristics, techniques, and acquisition protocols between the various institutions make the uniformity of data and subsequent comparison complex. Furthermore, most of the scientific studies are based on small population samples with a single-vendor MRI and common acquisition protocol. While this determines the homogeneity of the data, it could also lead to the presence of bias and overfitting [43]. Overfitting occurs when the model has a large number of input parameters, which may result in the inclusion of parameters that are not related to the disease but, for example, to noise. Therefore the model may perform well in the training (and even validation) phase but not on new data. This issue is usually addressed by a regularization process either during neural network training or through radiomic feature selection, which in both cases leads to a reduction of the inputs [5]. Other common limitations are that most studies on this topic are retrospective, and biological correlates are frequently lacking. Furthermore, the distribution of inputs can shift with time, resulting in a loss in accuracy. These shifts can be related to the aging of the detecting systems (which may degrade in performance over time), modifications to the characteristics of our input in the real world, and changes in the statistical relationship between the various inputs over time; this is commonly referred to as “data drift” [44]. A further limitation of applying radiomics in the field of pediatric neuro-oncology might be in the different diagnostic approval methodologies for pediatrics and adults. Currently, many radiomics researchers apply methods designed for adult patients to pediatric populations. Hence, the use and development of models that take the advantage of radiomic features for age-specific tumor biomarkers should be emphasized.

## Conclusions

The use of artificial intelligence (AI) algorithms and radiomics opens new and important scenarios in the realm of personalized medicine. The amount of data that can be obtained from neuroimaging is immense, far exceeding that derived from simple visual analyses performed by radiologists alone. Hence, there is a pressing need for new collaborations with other specialized professionals, such as big data analysts and AI specialists.

Most of the studies published in this field on the use of ML for the evaluation of radiomic features in p-CNS focused mainly on tumor characterization, especially those of the PF. Moving forward, it would be beneficial to deepen the use of the above-described techniques for the staging, treatment, and prognosis of these patients. Despite the limitations set out above, we believe that radiomics and AI algorithms have the potential to transition from their limited use in the research field to clinical applications in the diagnosis, treatment, and follow-up of small patients with brain tumors.

## Data Availability

Not applicable.
